# Role of gut microbiota in functional constipation

**DOI:** 10.1093/gastro/goab035

**Published:** 2021-08-06

**Authors:** Shengsheng Zhang, Ruixin Wang, Danyan Li, Luqing Zhao, Lixin Zhu

**Affiliations:** 1 Digestive Disease Center, Beijing Hospital of Traditional Chinese Medicine, Capital Medical University, Beijing, P. R. China; 2 Department of Colorectal Surgery, Guangdong Institute of Gastroenterology, Guangdong Provincial Key Laboratory of Colorectal and Pelvic Floor Diseases, The Sixth Affiliated Hospital of Sun Yat-sen University, Guangzhou, Guangdong, P. R. China

**Keywords:** gut microbiota, functional constipation, bile acids, SCFA, serotonin, traditional Chinese medicine

## Abstract

Functional constipation (FC) is common, yet the etiology is not clear. Accumulating evidence suggests an association between FC and abnormal gut microbiota. The relationship between the gut microbiota and the gut transit is likely bidirectional. This review summarizes the current evidence regarding the impact of gut microbiota on the pathogenesis of FC. By modulating the colonic motility, secretion, and absorption, gut microbiota may contribute to the development of FC through microbial metabolic activities involving bile acids, short-chain fatty acids, 5-hydroxytryptamine, and methane. In support of the key roles of the gut microbiota in FC, treatment with probiotics, prebiotics, synbiotics, and traditional Chinese medicine often result in compositional and functional changes in the gut microbiota. Further studies on the pathogenesis of FC and the therapeutic mechanism of microecological agents will provide a knowledge base for better management of FC.

## Introduction

**Figure 1.**
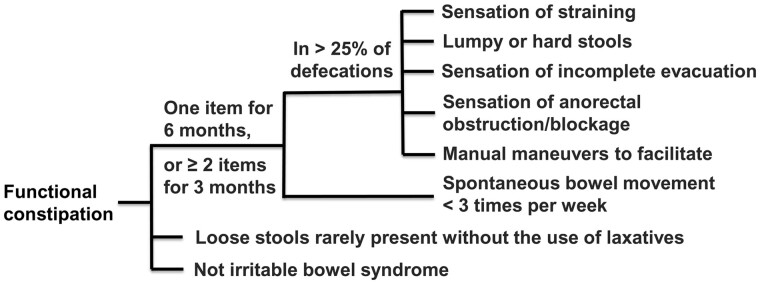
Diagnosis of functional constipation according to the Rome IV criteria.

Functional constipation (FC) refers to constipation without an organic etiology [1, 2]. Patients with FC have symptoms of predominantly difficult, infrequent, or a feeling of incomplete defecation, which may be accompanied by abdominal pain and bloating. FC has a significant impact on patients’ quality of life. According to the Rome IV criteria, to diagnose FC [3] (Figure 1), the patient must have one of the following conditions for more than 6 months and have two or more of the following conditions within the last 3 months: (i) sensation of straining during >25% of defecations, (ii) lumpy or hard stools of >25% of defecations (Bristol stool type 1 and 2) [4], (iii) sensation of incomplete evacuation during >25% of defecations, (iv) sensation of anorectal obstruction/blockage during >25% of defecations, (v) manual maneuvers for >25% of defecations, and (vi) fewer than three spontaneous bowel movements per week. In addition, diagnosis of FC should meet the requirement that loose stools are rarely present without using laxatives and that irritable bowel syndrome (IBS) is not diagnosed at the same time.

A recent demographic survey of 6,300 cases from three countries showed that the prevalence of FC was 6.9% (95% confidence interval [CI], 5.8%–8.0%) in the USA, 7.9% (95% CI, 6.7%–9.1%) in Canada and 8.6% (95% CI, 7.4%–9.9%) in the UK, according to the Rome IV criteria [5]. Globally, the prevalence of FC from 1947 to 2010 was 14% (95% CI, 12.0%–17.0%) according to the Rome I, II, III criteria or another informal diagnostic standard. South Asia and East Asia had the lowest prevalence of 11% (95% CI, 7.0%–15.0%), while South America had the highest prevalence of 18% (95% CI, 15.0%–22.0%). Caution needs to be exercised in comparing the incidences of FC in different countries since the diagnosis criteria and the methods for data collection may differ among studies. FC is positively associated with age and more frequently occurs in people who are >60 years old [6]. The pathophysiology of FC remains unknown, but it is generally considered to be multifactorial. Recognized pathophysiological factors include genetic traits; lifestyle including diet, fluid intake, physical activity; colonic dismotility; psychological factors such as anxiety and depression; and the gut microbiota, which is the main focus of this review. Traditionally, three types of FC have been recognized: normal-transit constipation (NTC), slow-transit constipation (STC) and rectal-evacuation disorders [7]. The majority of FC patients have NTC (65%), followed by evacuation disorder (30%) and STC (5%) [8].

The first choices for FC treatment are nonpharmacological interventions including education on toileting posture and behavior, dietary recommendations, and regular physical activity [9]. Ohlsson and Manjer [10] showed that the lack of exercise and regular diet habit are independent risk factors for gastrointestinal symptoms in patients with functional gastrointestinal diseases. Traditional pharmacological treatments include osmotic laxatives and stimulant laxatives. Polyethylene glycol (PEG), the most frequently used osmotic laxative for FC, increases fecal volume and promotes intestinal peristalsis. Many studies have shown that PEG increases the frequency of defecation and has fewer side effects [11]. Therefore, PEG can be used as a long-term first-line treatment. In contrast, although stimulant laxatives can make a fast improvement in stool consistency and frequency, they should not be used for >4 weeks considering the possible adverse effects [12]. New therapeutic agents, including prosecretory agents (e.g. linaclotide), serotonergic agents (e.g. 5-hydroxytryptamine 4 agonist), cholinesterase inhibitors (e.g. pyridostigmine), and bile-acid (BA) regulators (e.g. elobixibat) etc. may improve FC symptoms by promoting colon secretion and enhancing gastrointestinal motility [9]. Other treatment options with evidence for efficacy include biofeedback therapy [13], transanal irrigation [14], surgical interventions [15, 16], and neuromodulation [14]. Despite all these intervention options, 40% of pediatric [17] and more than half of adult FC patients [18–20] are dissatisfied with the treatments due to the lack of efficacy and adverse effects. Therefore, new management strategies have been explored. One of the possible intervention targets is the gut microbiome, which was supported by the observation that interventions with probiotics, prebiotics, and fecal-microbiota transplantation improved colonic transit and defecation frequency [21–23]. Herein, we summarize the current knowledge on the potential contribution of the gut microbiota in the pathogenesis of FC.

### Intestinal flora characteristics of patients with FC

Zoppi *et al*. [24] pioneered the study of the gut microbiota in FC using culture-based microbiological methods in 1998. They reported that constipation in children was associated with increased abundance in clostridia and bifidobacteria in the gut compared to healthy controls. Later, Khalif *et al*. [25] conducted a similar study with adult patients, still using culture-based microbiological methods, and reported that the abundances of *Bifidobacterium* and *Lactobacillus* were lower in constipated patients than in the controls. The opposite observations regarding the abundance of bifidobacteria may be explained by the pathophysiological differences between pediatric and adult patients. It is also important to note that culture-based methods tend to cause inaccurate observations in microbiota study because: (i) many species are not cultured, (ii) strict anaerobes die in an oxygenated environment and therefore tend to be underestimated, and (iii) *in vitro* culture changes the original structure of the microbiota.

In around 2015, Kim *et al*. [26] studied the microbiota of FC using a culture-independent method: the quantitative real-time polymerase chain reaction method. They reported that *Bifidobacterium* and *Bacteroides* species were decreased in the feces of FC. Although the methodology that Kim *et al*. [26] used is one large step more advanced than those reported in most studies on this topic, we have now progressed to the era of high-throughput sequencing and we conducted the first 16S rRNA sequencing study of the gut microbiota with adolescent FC patients [27]. Because we excluded those patients treated with antibiotics or proton-pump inhibitors, which are known to impact the gut microbiota, we were able to identify significant changes in the gut microbial composition of FC at every taxonomic level, with a relatively small sample size. At the genus level, the microbiota of FC exhibited a decreased abundance of *Prevotella* and increased abundance of *Coprococcus*, *Ruminococcus*, *Blautia*, *Anaerotruncus*, and *Clostridium*. *Prevotella* encodes a large set of enzymes for fiber metabolism [28] and is known for its association with dietary fibers [29]. Therefore, the decreased abundance of *Prevotella* in FC is consistent with the observation that FC patients usually have a low-fiber diet [30, 31]. In contrast to the findings of previous studies, conventional probiotic genera *Lactobacillus* and *Bifidobacterium* exhibited a trend for increased abundance in FC. At the community level, increased species richness was observed in the gut of FC, likely because of the prolonged incubation time of the gut microbiota in the presence of FC.

Recently, Mancabelli *et al*. [32] examined the gut microbial composition of adult FC patients using both the 16S rRNA sequencing and the whole-genome sequencing methods. Their 16S rRNA sequencing data indicated that the gut microbiota of FC patients was depleted of *Bacteroides*, *Roseburia*, and *Coprococcus 3*, which would predict a decreased level of short-chain fatty acid (SCFA) production. However, their whole-genome sequencing data did not validate this functional change.

Apparently, at this time, there is no consensus on the gut microbial structure characteristic of FC patients. Inconsistent observations may have been the consequences of the cultural and demographical differences of the study cohorts, different analysis techniques, and possible evolution of the disease over time. Table 1 summarizes several typical studies on the structural change in the gut microbiota in patients with FC.

**Table 1.** Structural changes in gut microbiota in functional constipation

Reference	Year	Methods	Inclusion criteria	Patients	Controls	Outcomes
Zoppi *et al.* [24]	1998	Microbial culture	Stool frequency less than one per 48 h and hard stool consistency	Children (*n* = 28, mean age 9.5 years)	Children (*n* = 14, mean age 7.9 years)	*Bifidobacteria*↑^*^Lactobacilli↑ *Bacteroides*↑ *Clostridia*↑^*^
Khalif *et al.* [25]	2005	Microbial culture	Rome II	Adults (*n* = 57, mean age 42.2 years)	Adults (*n* = 25)	*Bifidobacterium*↓^*^Lactobacillus↓ *Bacteroides*↓ *Clostridium*↓
Kim *et al.* [26]	2015	qRT-PCR	Rome III	Adults (*n* = 30, mean age 35 years)	Adults (*n* = 30, mean age 32 years)	*Bifidobacterium*↓^*^Lactobacillus↓ *Bacteroides*↓^*^Clostridium↓
Zhu *et al.* [27]	2014	16S rRNA	Clinical practice guideline developed by the North American Society for Pediatric Gastroenterology, Hepatology and Nutrition	Children (*n* = 8, mean age 11.8 years)	Children (*n* = 14, mean age 13.2 years)	*Prevotella*↓^*^Coprococcus↑^*^Ruminococcus↑^*^Blautia↑^*^Anaerotruncus↑^*^Clostridium^*^↑
Mancabelli *et al.* [32]	2017	16S rRNA and shotgun metagenomics	Rome III	Adults (*n* = 68, mean age 44 years)	Adults (*n* = 44, mean age 39 years)	*Bacteroides*↓^*^Roseburia↓^*^Coprococcus 3↓^*^ Faecalibacterium↑^*^

* *P *<* *0.05.

The relationship between the gut microbiota and the gut transit is likely bidirectional. Prolonged colonic transit in FC may facilitate the amplification and colonization of slow-growing species, leading to profound structural and functional alterations of the entire microecology. On the other hand, environmental factors may cause alterations in the gut microbiota, which, in turn, may contribute to the pathogenesis of FC through microbial metabolic activities.

### The contribution of intestinal flora in the pathophysiological mechanism of FC

**Figure 2.**
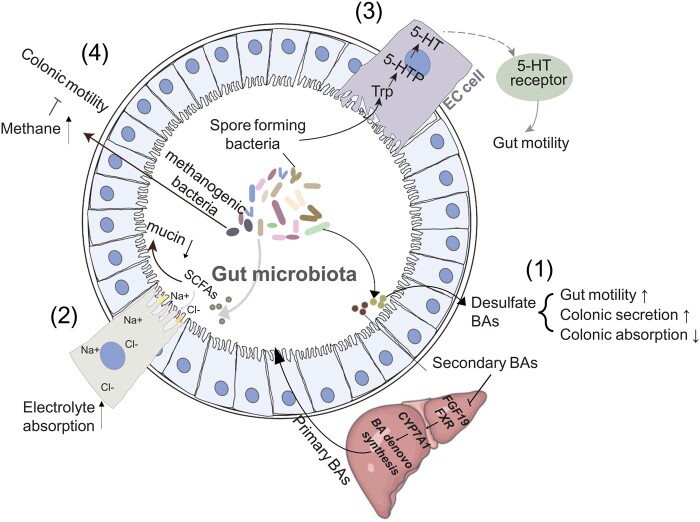
Potential molecular mechanisms for the gut microbiota that contribute to the pathogenesis of functional constipation (FC). (1) Bile acids (BAs) stimulate bowel movement and colonic secretion and suppress colonic absorption. The gut microbiota impacts these processes by regulating the BA levels, as well as the sulfation of BAs, which abolishes the effects of BAs on colonic transit. (2) Elevated levels of short-chain fatty acids (SCFAs), the major group of microbial fermentation products, stimulate electrolyte absorption and suppress mucin secretion, thus contributing to the pathogenesis of FC. The effect of SCFA on colonic motility is a controversial topic. (3) 5-hydroxytryptamine (5-HT) stimulates colonic motility. The gut microbiota regulates the level of 5-HT through several mechanisms and thereby affects the pathogenesis of FC. (4) Methane produced by the gut microbiota reduces bowel movement. 5-HTP, 5-hydroxytryptophan.

The current hypothesis is that the gut microbiota contributes to the pathogenesis of FC. This was supported by the observations that many risk factors of FC including age, diet, obesity, and stress have a large impact on the gut microbiota [33–35]. Thus, it is speculated that these risk factors may cause FC through mechanisms involving altered gut microbiota. The underlying mechanisms are the focus of the current review (Figure 2).

### Mechanisms involving BAs

Primary BAs are initially produced in the liver. Normally, most BAs are reabsorbed into the small intestine while ∼5% may arrive at the colon, where primary BAs are deconjugated and modified into secondary BAs by the gut microbiota [36, 37].

BAs may participate in the pathophysiology of FC through their effect on intestinal motility and colonic fluid transport. BAs are known to stimulate the release of 5-hydroxytryptamine (5-HT) and calcitonin gene-related peptide from enterochromaffin cells and intrinsic primary afferent neurons by activating the G protein-coupled BA receptor (TGR5), leading to the bowel peristaltic reflex [38, 39]. Several studies on FC patients treated with an ileal BA transporter inhibitor (elobixibat) demonstrated a causal relationship between elevated BA level and improved colonic transit [40–43].

There are direct and indirect mechanisms for BAs to stimulate fluid transport. BAs stimulate Cl^−^ secretion and inhibit Na^+^ absorption from colonic epithelial cells through regulation of the ion pumps, exchangers, and transporters [44]. In addition, BAs may indirectly stimulate colonic secretion through their effect on local neurons [45] and immune cells [46].

Animal studies suggest that it is the deconjugated BAs that impact colonic transit. BAs are conjugated with glycine or taurine before being secreted from hepatocytes. After arriving at the colon, BAs are deconjugated by bacterial bile salt hydrolase before further modification. Microbial transplantation studies in mice indicated that altered microbiota affects gastrointestinal transit, through its impact on BA deconjugation [47, 48]. It is noteworthy that the microbial metabolisms of BAs are different between mice and humans, thus the mice studies remain to be validated in humans.

Several mechanisms have been proposed to explain how BAs mediate the microbial effects on FC. First, the gut microbiota may affect gastrointestinal transit through its regulation of BA synthesis. The gut microbiota is equipped with enzymes for the production of secondary BAs, which may suppress the FGF19- and FXR-mediated signaling, leading to the induction of CYP7A1 [49, 50], and consequently elevated *de novo* BA synthesis and improved colonic transit. To test this hypothesis, an integrated study on the colonic BA profiles, the abundances of BA metabolizing bacteria, and the cell biology of the colonic epithelium is required.

Another mechanism for the gut microbiota to influence FC is through its impact on BA sulfation, which abolishes the effect of the BAs on fluid transport [51, 52]. BA sulfation likely occurred in hepatocytes, while colonic bacterial bile salt sulfatase may desulfate BA, empowering its effect to stimulate colonic secretion and transit, and consequently improve colonic fluid transport [53]. Future study may characterize the abundances of sulfated BAs in the gut of patients with FC and its association with sulfatase-encoding bacteria.

### Mechanisms involving SCFAs

SCFAs including acetate, propionate, and butyrate are the major fermentation products of the gut microbiota. Elevated levels of SCFAs are found in the stools of FC patients. Jalanka *et al*. [54] reported that the level of fecal acetate in FC patients was 2.2-fold higher than that in healthy controls, and that the levels of acetate, butyrate, and propionate were associated with the transit time in constipated patients. Iso-butyrate levels were also significantly higher in FC subjects than in healthy subjects [55].

SCFAs may contribute to the pathogenesis of FC by regulating colonic electrolyte absorption and secretion. SCFAs, especially butyrate, stimulate electrolyte absorption. The stimulation of Na^+^ and Cl^−^ absorption by mucosal butyrate was greater than that by propionate and acetate [56]. The effect of butyrate on mucin secretion takes a biphasic mode: butyrate stimulation of mucin section peaks at 5 mM of butyrate. At concentrations of >5 mM, butyrate is inversely correlated with mucin section [57]. These results consistently support the roles of elevated SCFAs in increased electrolyte absorption and decreased mucin secretion. On the other hand, the effect of SCFAs on colonic motility is controversial. In some studies, SCFAs increased colonic motility in rats [58, 59], whereas other studies reported opposite observations [60, 61]. Several gaps and pitfalls are to be addressed to understand the role of SCFAs in FC. First, SCFAs measured in the published studies represent what were not absorbed, whereas the relevant SCFAs are those in contact with the SCFA receptors. Second, the working concentrations in different species likely vary and caution is needed when interpreting observations made in studies using SCFA concentrations that are far higher than physiological concentrations. Third, the long-term effects of SCFAs on the neural structure are important and need to be considered when interpreting the SCFA effects on colonic secretion, absorption, and motility [59].

Studies on the structure of the gut microbiota seem to support a role for SCFAs in the pathogenesis of FC. Butyrate-producing genera, *Roseburia* and *Faecalibacterium*, were increased in adolescent FC according to 16S rRNA sequencing studies [27, 62]. In a similar 16S rRNA sequencing study for adult FC patients, Mancabelli *et al*. [32] reported that *Faecalibacterium* is elevated in FC. However, their data indicated that other butyrate-producing bacteria *Roseburia* and *Coprococcus 3* were depleted in FC. Shotgun metagenomic sequencing by Mancabelli *et al*. [32] does not support altered SCFA production in FC, which may be explained by very small sample size. Further study integrating microbiome survey, metabolome analysis, colonic absorption, secretion, and colonic motility is warranted to understand the role of the gut microbiota in promoting FC through altered SCFA production.

### Mechanisms involving 5-HT

Produced by enterochromaffin cells, 5-HT is an abundant neurotransmitter in the enteric nervous system. Although it is a controversial subject, a significant amount of evidence suggests that 5-HT stimulates colonic motility through its receptors 5-HT3 and 5-HT4 [63]. For example, prucalopride, a highly selective agonist for serotonin 5-HT4 receptor, increases the number of bowel movements per week in adults with chronic FC [64]. Thus, the gut microbiota may impact the gut motility by regulating the production of 5-HT. Theoretically, the gut microbiota may regulate the production of 5-HT via several mechanisms. First, the gut microbiota influences the growth of colon enterochromaffin cells, suggested by the upregulation of 5-HT–positive enterochromaffin cells in germ-free rats [65]. In contrast to that of rats, the gut microbiota of mice and humans seem to have an opposite effect on 5-HT production. Mediated by microbial metabolites, indigenous spore-forming bacteria (Sp) from the mouse and human microbiota promote 5-HT biosynthesis from colonic ECs [66]. Independent studies have suggested that microbial metabolites BAs and SCFAs may induce the release of 5-HT from enterochromaffin cells [38, 39, 67].

A recent study suggests a different mechanism. Cao *et al*. [68] reported that the gut microbiota of FC patients upregulates the expression of serotonin transporter, which removes 5-HT from the gut. This causes decreased colonic transit and FC. It is noteworthy that the authors stated several times that they performed a 16S pyrosequencing study, but provided a description of illumina Miseq sequencing in the method section [68].

Finally, the gut microbiota may influence 5-HT production by regulating tryptophan metabolism in the gut [69]. For example, the gut microbiota may upregulate the production of indole and kynurenine from tryptophan, thereby reducing the substrate for the production of 5-HT and consequently leading to FC.

A fundamental gap in the study of 5-HT in FC is whether mucosal 5-HT is altered in FC patients. While some studies reported decreased 5-HT [64, 70], there were reports that the 5-HT level was not altered [71] or was increased in FC [72]. Vigorous studies are needed to validate the role of 5-HT in mediating the effect of gut microbiota on the pathogenesis of FC.

### Mechanisms involving methane

It has long been proposed that methanogenic gut microbiota causes constipation by reducing bowel movements [73]. The hypothesis has been supported by the observations that FC patients carry gut microbiota enriched with methanogenic bacteria [74, 75]. In line with this, in patients with constipation-predominant IBS, treatment with antibiotics reduced the methanogenic bacteria in the gut microbiota and led to improved symptoms [76]. However, it is worth noting that no control was used in this retrospective study. On the other hand, one study reported that in patients with FC, methane production was associated with the gut microbial composition, but not with constipation or colonic transit [77]. Caution is also required to interpret these data as all of the 25 patients with constipation included 13 FC, 6 IBS with constipation, and 6 mixed IBS. Intervention studies with more strict inclusion criteria and a larger sample size are needed to clarify the role of methanogenic bacteria in FC.

### FC treatments targeting the gut microbiota

In support of the roles of the gut microbiota in the pathogenesis of FC, various microbial interventions including probiotics, prebiotics, synbiotics, and traditional Chinese medicine (TCM) have shown beneficial effects on FC. In addition, some of the intervention studies support the mechanisms discussed above.

### Probiotics

Probiotics, the most widely used microecologics, are effective in treating a wide variety of diseases by regulating the immune response, preventing the colonization of pathogens, improving gut barrier function, and reducing stress and anxiety, etc. [78]. Although individual studies have reported varied efficacies [79, 80], an earlier systemic review and meta-analysis of randomized–controlled trials indicated that probiotics may improve the whole-gut transit time, stool frequency, and stool consistency; *Bifidobacterium lactis* showed better efficacies than *Lactobacillus casei Shirota* [81]. A more recent systemic review and meta-analysis arrived at a similar conclusion that probiotics increase stool frequency and decrease intestinal transit time in FC patients [82]. Most recently, a meta-analysis of randomized–controlled trials of probiotics on FC concluded that probiotics consisting of multispecies, not single species such as *B.* *lactis* or *B. longum* alone, significantly reduced the whole-gut transit time, increased the defecation frequency, improved stool consistency, and decreased bloating [83]. It is worth noting that probiotics are likely to have a greater effect on the small bowel than on the colon, as the small bowel has far fewer competing bacteria. One study showed that probiotics reduced both the small-bowel transit time and the colonic transit time [84]. It is possible that the shortened small-bowel transit would increase the inflow to the colon and would consequently accelerate colonic transit.

However, similar studies with pediatric patients do not support the efficacy of probiotics on pediatric FC [85, 86]. The difference between adult and pediatric patients with FC in response to probiotics may be related to different microbial compositions in adult and pediatric subjects: while adult patients with FC exhibited a decreased abundance of *Bifidobacterium* [26], adolescent patients with FC exhibited a trend for elevated abundance of *Bifidobacterium* and *Lactobacillus* [27].

To explore the different effects of probiotic strains in FC, Wang *et al*. [87] treated constipated mice with *B.* *longum*, *B. infantis*, *B. animalis*, *B. bifidum*, *B. adolescentis*, and *B. breve*, respectively. They observed that *B. longum*, *B. infantis*, and *B. bifidum* were the most effective strains to relieve constipation. The improved symptoms were attributed to increased abundance of *Lactobacillus* and reduced levels of pathogenic bacteria (*Alistipes*, *Odoribacter*, and *Clostridium*). It is important to note that a randomized, double-blind, placebo-controlled probiotics treatment trial on FC is rare. In one of these trials, Ibarra *et al*. [88] reported no difference between probiotics and the placebo in primary analysis, but in a post hoc analysis, they reported that *B.* *animalis subsp. lactis* HN019 (HN019) increased the frequency of spontaneous defecations and reduced the degree of straining in FC patients.

### Prebiotics

Prebiotics refers to non-digestible food ingredients that beneficially affect the host by selectively stimulating the growth and/or activity of one or a limited number of bacteria in the colon [89]. Recently, a randomized placebo-controlled trial of prebiotics for the treatment of FC was reported. UG1601, composed of inulin, lactitol, and aloe vera gel, was used to treat female patients with mild chronic FC [90]. Although UG1601 seemed to be more effective than placebo in improving abdominal and fecal symptoms, statistical significance was not achieved. Other interesting observations include reduced levels of serum cluster of differentiation 14 (CD14) and lipopolysaccharide (LPS), and increased abundance of *Roseburia hominis*, a butyrate-producing bacterium, upon UG1601 treatment.

D-tagatose is a monosaccharide often used as a food supplement. Liang *et al.* [91] found that high-dose d-tagatose restored the gastrointestinal transit rate of constipated mice induced by loperamide to a similar level of that of control mice, and improved the overall defecation condition including fecal weight, fecal number, and time to the first black-stool defecation. These therapeutic effects were attributed to the increased levels of excitatory neurotransmitters (Ach and SP) and the reduced level of inhibitory neurotransmitters (NO). The therapeutic mechanisms of d-tagatose may also involve the gut microbiota as the prebiotic therapy restored the composition of the intestinal flora.

Similarly, partially hydrolysed guar gum (HHGG), a fiber supplement, was shown to increase the fecal water content and enhance the small-intestinal transit of loperamide-induced constipated mice [92]. The therapeutic effects may be mediated by the gut microbiota as the prebiotics caused significant changes in the gut microbiota and elevated production of SCFAs.

Another popular prebiotics, β-glucan, is a polysaccharide widely found in yeast, fungus, and plants. Chen *et al*. [93] used the β-glucan extracted from bread yeast cells to treat loperamide-induced constipated mice and found enhanced intestinal motility. The pharmacological effect of β-glucan may be mediated by the enhanced expression of epithelial tight junction proteins (zonula occludens-1 and mucin-2) and neurotransmitters (acetylcholinesterase and serotonin). The gut microbiota was likely involved in the therapeutic effect of β-glucan as it restored the intestinal flora of the constipated mice toward a normal composition.

Although efficacies were shown with the loperamide-induced mice model, these prebiotics remain to be validated in randomized, double-blind, placebo-controlled trials.

### Synbiotics

Synbiotics are combinations of probiotics and prebiotics, which may exhibit synergistic effects of both components [94]. In a pilot randomized, double-blind, controlled trial of a small sample size, synbiotic supplement Psyllogel Megafermenti improved defecation and decreased ITT [95]. However, in another randomized, double-blind, placebo-controlled trial with a larger sample size, no significant effect was found for a synbiotic composed of *B.* *lactis* BB12, *L.* *plantarum* LP01, and inulin-oligofructose [96]. A more recent trial using a combination of polydextrose and *L.* *helveticus* found beneficial effects on intestinal transit and fecal pH, but no significant advantage was found with this synbiotic compared with *L. helveticus* alone [97]. Perhaps different types of synbiotics have different therapeutic efficacies on FC. More clinical trials are needed to identify effective synbiotics and to confirm the therapeutic effects.

### TCM

Several TCM herbs and formulations are effective for FC. The hemp seed soft capsule (HSSC) was developed from the ancient traditional prescription ‘hemp seed pill’, which consists of *Semen Cannabis*, *Magnolia officinalis*, *Fructus Aurantii Immaturus*, *Radix Paeoniae Alba*, *Almond*, and *Rheum rhabarbarum*. As a representative prescription of TCM in the treatment of constipation [98], the hemp seed pill has been known to improve colonic secretion and transit [99]. With loperamide-induced constipated rats, Lu *et al.* [100] showed that HSSC increased the fecal wet weight and water content, which was attributed to the combined actions of cAMP-dependent and Ca^2+^-dependent Cl^−^ channels, NKCC, Na^+^-HCO3^−^ co-transporter, or Cl^−^/HCO3^−^ exchanger.

Recently, the gut microbiota has been often reported to participate in the therapeutic effects of these herbs and formulations. Invented in the Qing Dynasty ∼300 years ago, Zengye decoction (ZYD) has been used to cure ‘dryness’ by promoting the production of body fluids according to TCM theory. Liu *et al*. [101] examined the effect of ZYD on the gut microbiota of constipated rats. They found that ZYD restored the composition of the gut microbiota toward a normal state by reducing the abundance of *Helicobacteraceae*, *Desulfovibrionaceae*, *Ruminococcaceae*, *Lactobacillaceae*, *Prevotellaceae*, and *Dorea*, while increasing the abundance of *Aeromonadaceae*, *Oxalobacteraceae*, *Veillonellaceae*, *Clostridiaceae*, and *Roseburia*. Metabolomic analysis revealed that ZYD caused microbial changes in the metabolism of energy, amino acids, methane, and glutathione.

Records of mulberry fruit for the treatment of constipation and other digestive diseases date back to 200 BC. According to TCM theory, mulberry fruit can be used to treat ‘yin’ deficiency. Hu *et al.* [102] used the mulberry fruit to treat diphenoxylate-induced constipated mice and found that the treatment increased the fecal water content, shortened the first red-stool defecation time, and increased the gastric-intestinal transit rate. The mulberry-fruit treatment caused alterations in the gut microbiota, with increased abundance of *Lactobacillus*, *Bifidobacterium*, and *Eubacterium*, and decreased abundance of *Helicobacter*, *Alloprevotella*, and *Prevotellaceae*. The compositional change in the microbiota was accompanied by decreased expression of aquaporin genes (Aqp3, Aqp4, Aqp8, and AqP9), reduced levels of inhibitory neurotransmitters, and increased levels of excitatory neurotransmitters and SCFAs, suggesting a therapeutic mechanism whereby mulberry fruit causes a change in the microbiota, leading to changes in microbial metabolites, which, in turn, improves colonic motility and secretion.

Sennoside A, the main active constituent of Da-Huang-Gan-Cao-Tang (Daiokanzoto, DKT), is converted by microbial β-glucosidases to generate rheinanthrone, the molecule with laxative activity. Because of the close connection between sennoside A and the gut microbiota, it was hypothesized that the therapeutic effect of sennoside A depends on the composition and function of the gut microbiota, which was proved in mice carrying different types of gut microbiota. Takayama *et al.* [103] proposed that different types of gut microbiota represent different ‘patterns’ defined by TCM and therefore they established a model to investigate the biological mechanisms behind the personalized medicinal practices in TCM. In DKT, sennoside A is mainly found in its herbal component of rhubarb. In fact, many other TCM formulations for the treatment of FC have a component of rhubarb, which was shown to increase intestinal secretion and improve stool consistency [104].

TCM usually takes the form of a complex composition and is multi-targeting. Understanding the links between changes in the composition of the intestinal flora, the altered gene expression of the intestines, and the metabolites produced after TCM therapy requires further investigation.

## Conclusions

Microecological imbalance is an important feature in FC, which may contribute to the pathogenesis via multiple mechanisms mediated by microbial metabolites including BAs, SCFAs, 5-HT, and methane. The therapeutic effects of probiotics, prebiotics, synbiotics, and TCM often involve compositional and functional changes in the gut microbiota. Further studies on the pathomechanisms of FC and the therapeutic mechanisms of microecological agents will provide a knowledge base for better management of FC patients. Given the very different diet and the gut microbiota of laboratory animals compared to those of humans, understanding the therapeutic efficacy and the mechanisms of microecological agents may require adequately powered mechanistic clinical trials with FC patients.

## Authors’ Contributions

L.Q.Z. and L.X.Z. conceived of this work and designed the outlines of this review. S.S.Z. and L.X.Z. selected the references and prepared the first draft. All authors critically revised the manuscript and approved the final version for submission.

## Funding

This work was supported by the Clinical Medicine Development Project of Beijing Municipal Administration of Hospitals [ZYLX201411], the Beijing Nova Program [Z201100006820119] from Beijing Municipal Science & Technology Commission, the Beijing Science and technology project [z181100001718218], the National Natural Science Foundation of China [81770571], the Guangdong Province ‘Pearl River Talent Plan’ Innovation and Entrepreneurship Team Project [2019ZT08Y464], and the National Key Clinical Discipline of China.
